# Supplementary material for: The role of TyG index as a predictor of all-cause mortality in hospitalized patients with acute pancreatitis: a retrospective study utilizing the MIMIC-IV database
Source: PLoS One. 2025 Mar 25;20(3):e0308994. doi: 10.1371/journal.pone.0308994 (PMC11936218; doi:10.1371/journal.pone.0308994)
Supplement: S1 Table — (DOCX) [file pone.0308994.s001.docx]

**Table S1.** missing number for risk variables and outcome variables

| Risk Variables | Missing number (%) |
| --- | --- |
| Age (years) | 0 |
| Height (cm) | 6 (0.62%) |
| Weight (kg) | 7(0.94%) |
| BMI | 13 (1.38%) |
| Gender: male | 0 |
| SOFA | 0 |
| APS III | 0 |
| SAPS II | 0 |
| SIRS | 0 |
| Commorbidities |  |
| Heart failure | 0 |
| Respiratory failure | 0 |
| AKI_48h | 0 |
| Diabetes | 0 |
| Hypertension | 0 |
| Sepsis | 0 |
| Laboratory tests |  |
| WBC, K/uL | 1 (0.12%) |
| Platelet, K/uL | 6 (0.58%) |
| Hemoglobin, g/dL | 5 (0.51%) |
| Sodium, mEq/L | 0 |
| Serum creatinine | 0 |
| TG, mg/d | 0 |
| FBG, mg/dL | 0 |
| TyG index | 0 |
| Lactate,mg/dl | 0 |

Abbreviation: TyG index, triglyceride glucose index; BMI, body mass index; AKI_48hr: Acute Kidney Injury within 48 h; SOFA, sequential organ failt; SIRS:Syure assessmenstemic Inflammatory Response Syndrome;APSIII, acute physiology score III; SAPSII, simplifed acute physiological score II; WBC, white blood cell; TG, triglyceride; FBG, fasting blood glucose;
