# Supplementary material for: The role of TyG index as a predictor of all-cause mortality in hospitalized patients with acute pancreatitis: a retrospective study utilizing the MIMIC-IV database
Source: PLoS One. 2025 Mar 25;20(3):e0308994. doi: 10.1371/journal.pone.0308994 (PMC11936218; doi:10.1371/journal.pone.0308994)
Supplement: Table S2 — (DOCX) [file pone.0308994.s002.docx]

**Table S2**  Characteristics and outcomes of participants categorized by TyG index

| **Variable** | **Total (n = 586)** | **Q1-3 (n = 439)** | **Q4 (n = 147)** | **P** |
| --- | --- | --- | --- | --- |
| Age, years | 56.00 (45.00 - 70.00) | 56.00 (41.00 - 69.50) | 57.00（43.00-70.00） | 0.994 |
| Gender, n (%) | 262 (44.71) | 198 (45.10) | 64 (43.54) | 0.741 |
| BMI, ,kg/m2 | 30.55 ± 5.97 | 30.38 ± 6.04 | 31.06 ± 5.72 | 0.232 |
| WBC, K/uL | 14.26 ± 8.57 | 14.23 ± 8.98 | 14.35 ± 7.24 | 0.878 |
| Platelet, K/uL | 217.33 ± 130.70 | 212.71 ± 125.16 | 231.09 ± 145.56 | 0.140 |
| Hemoglobin, g/dL | 11.57 ± 5.29 | 11.57 ± 5.94 | 11.55 ± 2.48 | 0.970 |
| Albumin,g/dL | 2.89 ± 0.56 | 2.87 ± 0.55 | 2.94 ± 0.58 | 0.196 |
| Sodium ,mEq/L | 138.04 ± 5.84 | 138.00 ± 5.75 | 138.16 ± 6.12 | 0.782 |
| Potassium,mEq/L | 4.10 ± 0.84 | 4.04 ± 0.73 | 4.29 ± 1.09 | 0.012 |
| Glucose, mg/dl | 156.23 ± 117.01 | 131.46 ± 59.15 | 230.20 ± 192.38 | <.001 |
| Lactate,mg/dl | 1.90 (1.30 - 3.10) | 1.83 (1.20 - 2.90) | 2.04 (1.40 - 3.41) | 0.037 |
| Fibrinogen, mg/L | 424.99 (309.26 - 518.92) | 424.00 (308.11 - 516.39) | 425.00 (312.70 - 530.53) | 0.391 |
| Anion gap,mEq/L | 16.14 ± 5.48 | 15.74 ± 5.27 | 17.32 ± 5.94 | 0.002 |
| Triglyceride,mg/dl | 438.55 ± 728.10 | 280.90 ± 193.23 | 909.35 ± 1309.30 | <.001 |
| Creatinine,mg/dL | 1.52 ± 1.81 | 1.54 ± 1.95 | 1.46 ± 1.32 | 0.639 |
| Amylase,IU/L | 311.61 ± 301.66 | 312.02 ± 296.87 | 310.37 ± 316.60 | 0.954 |
| SOFA | 5.00 (2.00 - 8.00) | 4.00 (2.00 - 8.00) | 5.00 (2.00 - 8.00) | 0.326 |
| APSIII | 47.00 (33.00 - 65.00) | 47.00 (33.00 - 65.00) | 47.00 (35.00 - 65.00) | 0.978 |
| SIRS | 3.00 (2.00 - 4.00) | 3.00 (2.00 - 4.00) | 3.00 (3.00 - 4.00) | 0.555 |
| SAPSII | 33.00 (24.00 - 45.00) | 33.00 (24.00 - 45.00) | 33.00 (25.00 - 46.00) | 0.885 |
| TyG index | 9.95 (9.20 - 10.57) | 9.60 (8.99 - 10.11) | 11.00 (10.75 - 11.29) | <.001 |
| Heart failure, n (%) | 74 (12.63) | 55 (12.53) | 19 (12.93) | 0.900 |
| AKI_48hr, n (%) | 339 (57.85) | 255 (58.09) | 84 (57.14) | 0.841 |
| Respiratory failure, n (%) | 229 (39.08) | 168 (38.27) | 61 (41.50) | 0.488 |
| Sepsis, n (%) | 161 (27.47) | 123 (28.02) | 38 (25.85) | 0.610 |
| Diabete, n (%) | 160 (27.3) | 98 (22.32) | 62 (42.18) | <.001 |
| HP, n (%) | 324 (55.29) | 230 (52.39) | 94 (63.95) | 0.015 |
| MV, n (%) | 237 (40.44) | 175 (39.86) | 62 (42.18) | 0.621 |
| CRRT, n (%) | 61 (10.41) | 44 (10.02) | 17 (11.56) | 0.596 |
| Hospital_Los,Day | 10.41 (6.61 - 18.06) | 10.04 (6.15 - 16.85) | 12.66 (7.75 - 21.33) | 0.002 |
| ICU_Los,Day | 3.32 (1.89 - 6.84) | 3.11 (1.85 - 6.04) | 4.20 (2.04 - 8.98) | 0.011 |
| Hospdead, n (%) | 113 (19.28) | 66 (15.03) | 47 (31.97) | <.001 |
| ICUdead, n (%) | 76 (12.97) | 39 (8.88) | 37 (25.17) | <.001 |

Abbreviation:BMI: Body Mass Index; WBC: White Blood Cell; AKI_48hr: Acute Kidney Injury within 48 h; SOFA: Sequential Organ Failure Assessment;APSIII, Acute Physiology Score III; SIRS:Systemic Inflammatory Response Syndrome;SAPSII: Simplifed Acute Physiological Score II; Hospital_Los,Day: Hospital Length of Stay; ICU_Los,Day: Intensive Care Unit Length of Stay;
