# Supplementary material for: The role of TyG index as a predictor of all-cause mortality in hospitalized patients with acute pancreatitis: a retrospective study utilizing the MIMIC-IV database
Source: PLoS One. 2025 Mar 25;20(3):e0308994. doi: 10.1371/journal.pone.0308994 (PMC11936218; doi:10.1371/journal.pone.0308994)
Supplement: S3Table — (DOCX) [file pone.0308994.s003.docx]

**Table S3**  Binary logistic regression analysis of the factors influencing all-cause death of the study population.

| **Variables** | **Z** | **OR (95%CI)** | **P** | **aZ** | **aOR (95%CI)** | **aP** |
| --- | --- | --- | --- | --- | --- | --- |
| Age | 3.61 | 1.02(1.01-1.03) | <.001 | 3.57 | 1.02(1.01-1.03) | <.001 |
| Gender,male | -0.44 | 0.92(0.65-1.31) | 0.657 |  |  |  |
| BMI | 2.08 | 1.03(1.01-1.06) | 0.037 |  |  |  |
| APSIII | 4.14 | 1.01(1.01-1.02) | <.001 | 2.88 | 1.01(1.01-1.02) | 0.004 |
| Platelet, K/uL | -0.94 | 1.00(1.00-1.00) | 0.349 |  |  |  |
| Hemoglobin, g/dL | -0.54 | 0.99(0.94-1.03) | 0.588 |  |  |  |
| Glucose, mg/dl | 3.42 | 1.01(1.01-1.01) | <.001 |  |  |  |
| Anion gap,mEq/L | 4.14 | 1.07(1.04-1.10) | <.001 | 2.57 | 1.05(1.01-1.09) | 0.010 |
| Lactate,mg/dl | 1.98 | 1.08(1.01-1.17) | 0.048 |  |  |  |
| Triglyceride,mg/dl | 2.00 | 1.01(1.01-1.01) | 0.046 |  |  |  |
| TyG Index | 6.00 | 1.81(1.49-2.19) | <.001 | 5.82 | 1.80(1.48-2.19) | <.001 |
| AKI_48hr, n (%) | -3.27 | 0.56(0.39-0.70) | 0.001 |  |  |  |
| Respiratory failure, n (%) | 1.29 | 1.26(0.89-1.80) | 0.196 |  |  |  |
| Sepsis, n (%) | 2.38 | 1.58(1.08-2.31) | 0.017 |  |  |  |
| Diabete, n (%) | 0.87 | 1.19(0.81-1.74) | 0.383 |  |  |  |
| Hypertension, n (%) | 1.15 | 1.23(0.87-1.75) | 0.248 |  |  |  |
| Heart failure, n (%) | -0.50 | 0.87(0.51-1.49) | 0.620 |  |  |  |

Abbreviation:BMI: Body Mass Index; AKI_48hr: Acute Kidney Injury within 48 h; APSIII, Acute Physiology Score III;
