# Supplementary figures and images for: The role of TyG index as a predictor of all-cause mortality in hospitalized patients with acute pancreatitis: a retrospective study utilizing the MIMIC-IV database
Source: PLoS One. 2025 Mar 25;20(3):e0308994. doi: 10.1371/journal.pone.0308994 (PMC11936218; doi:10.1371/journal.pone.0308994)

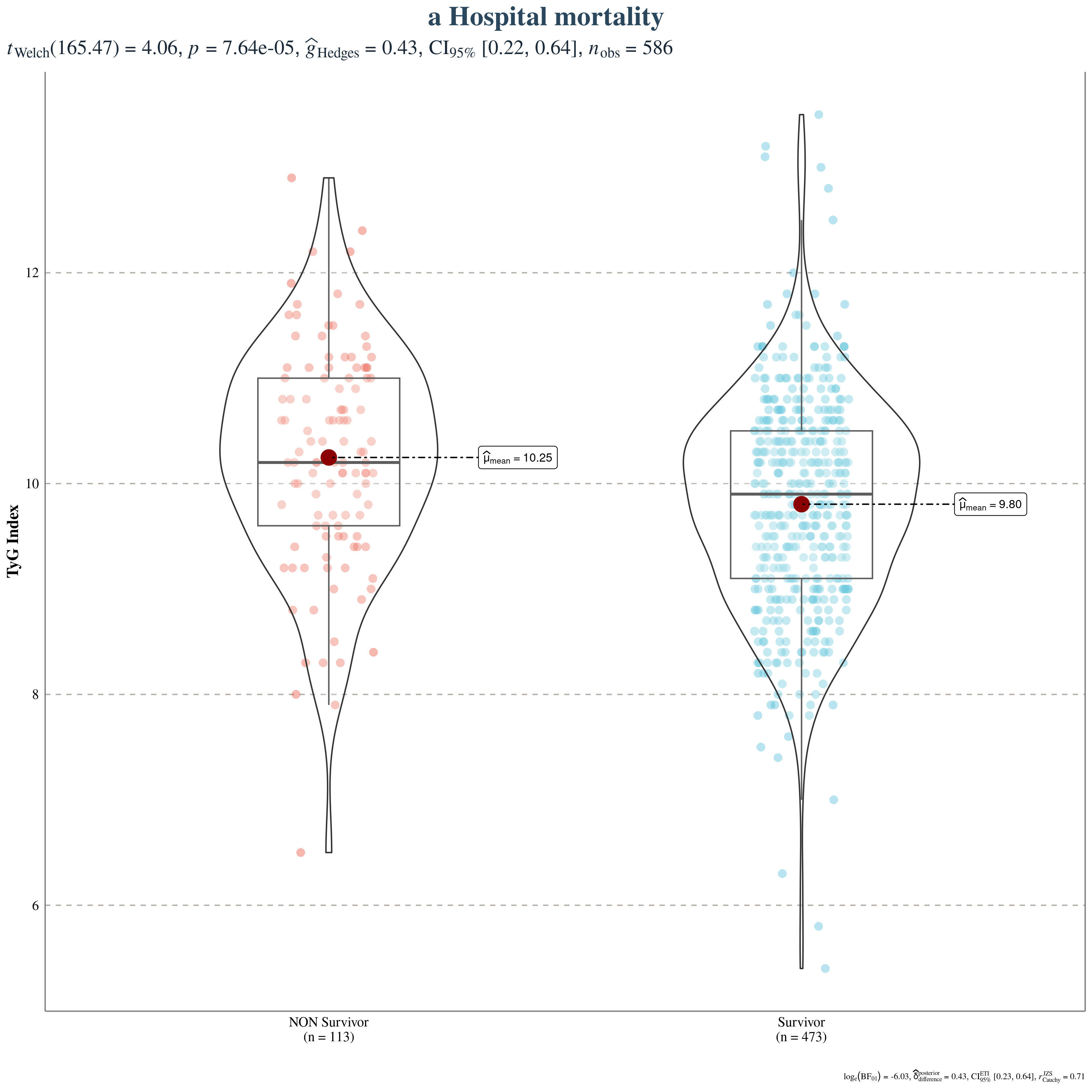

Supplement: S1 a Fig — (PNG) [file pone.0308994.s004.png]

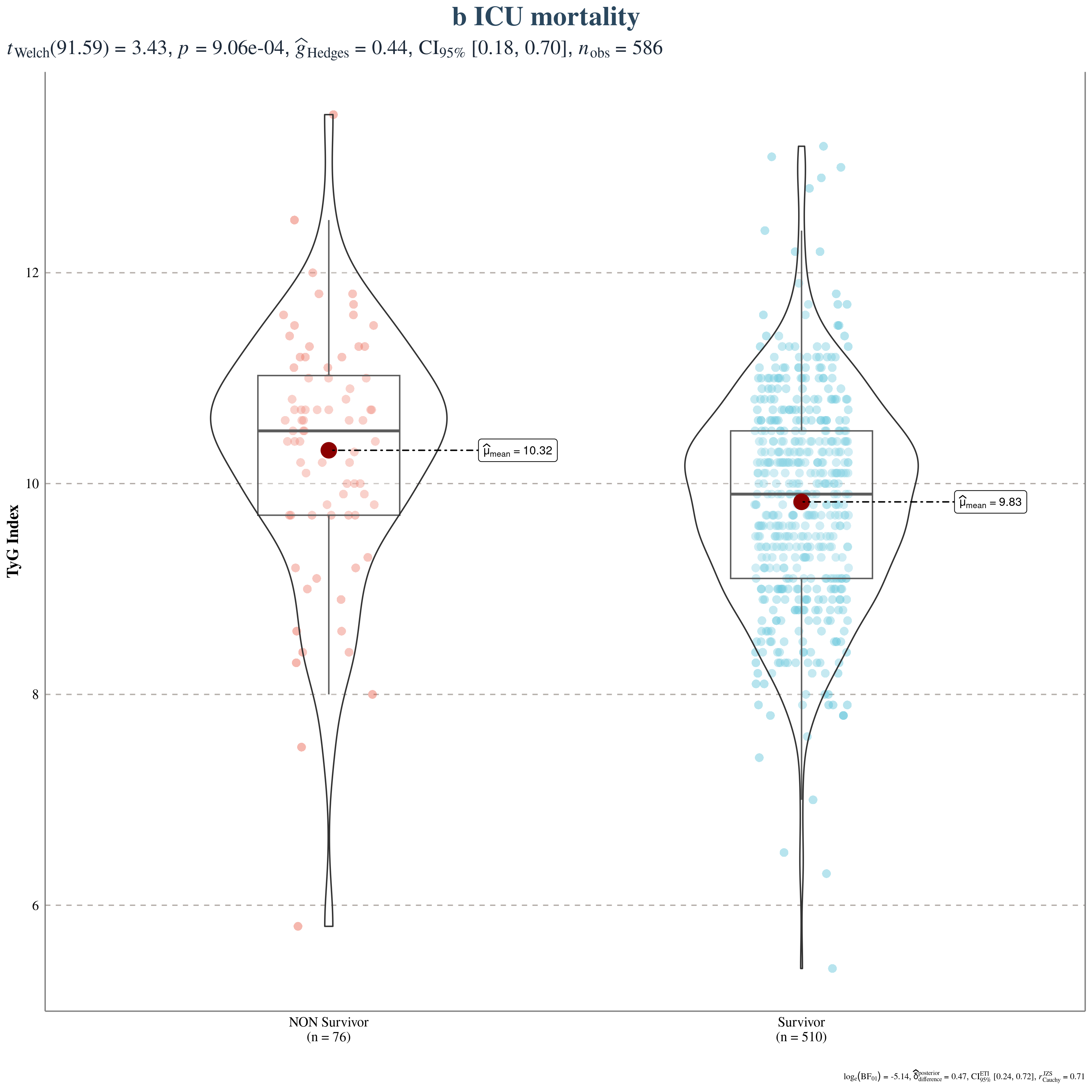

Supplement: S1 b Fig — (PNG) [file pone.0308994.s005.png]

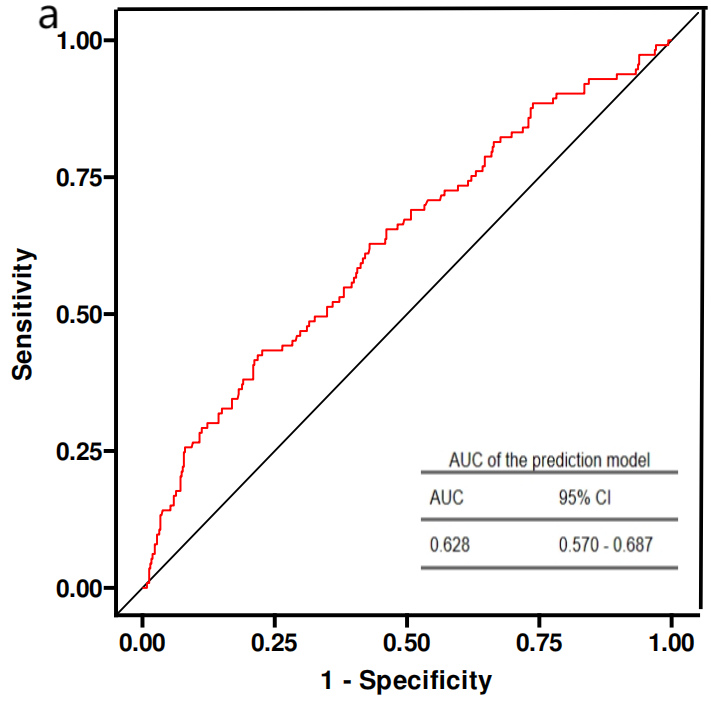

Supplement: S2 a Fig — (PNG) [file pone.0308994.s006.png]

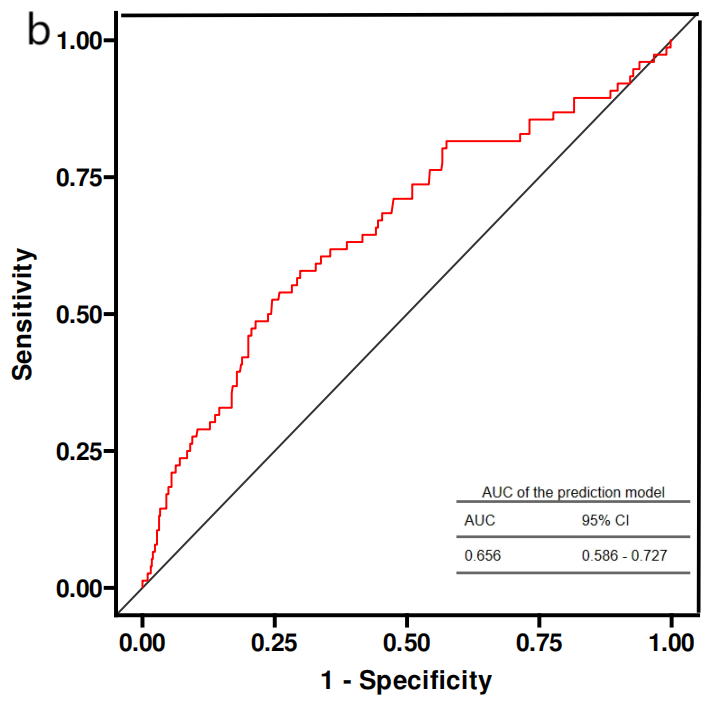

Supplement: S2 b Fig — (PNG) [file pone.0308994.s007.png]
