# Supplementary material for: The role of TyG index as a predictor of all-cause mortality in hospitalized patients with acute pancreatitis: a retrospective study utilizing the MIMIC-IV database
Source: PLoS One. 2025 Mar 25;20(3):e0308994. doi: 10.1371/journal.pone.0308994 (PMC11936218; doi:10.1371/journal.pone.0308994)
Supplement: Attachment — Submitted filename: Response to Reviewers.docx [file pone.0308994.s009.docx]

Response to reviewers

Dear Editor and reviewers,

Thank you for reviewing our manuscript and for the constructive comments, which greatly helped us to improve the manuscript (PONE-D-24-05736). We appreciate the promoting comments to our study, and we have accepted and revised as recommended in this revised manuscript. We highlighted all the revisions in yellow/red colour.

We would like to express our great appreciation to you and reviewers for comments on our paper. Looking forward to hearing from you.

Thank you and best regards.

The main corrections in the paper and the responds to the reviewer’s comments are as following:

**Reviewer #1:**

**Comment 1:****Selection Bias**: Given that this study is retrospective, I suggest elucidating the selection bias of patients due to the retrospective nature of the study.

**Response:** It is really true as Reviewer suggested that the selection bias of patients should be explained.There is a potential selection bias to this retrospective study. Therefore, more prospective studies with a larger cohort of subjects are needed to support the present findings. We have mentioned this in the last paragraph of Discussion marked in yellow.f

**Comment 2:****Accuracy of the TyG Index**: The TyG index was calculated from initial measurements of glucose and triglycerides. I suggest clarifying how these measurements accurately reflect insulin resistance or the overall metabolic state throughout the patient's hospitalization. Additionally, it is important to consider that acute states of pancreatitis can falsely lower triglyceride levels, which can be a significant bias. The inflammatory state of pancreatitis can correspond to a Systemic Inflammatory Response Syndrome (SIRS), which can elevate other markers and, by itself, increase glucose levels and temporarily reduce insulin resistance.

**Response:** We have reviewed the relevant literature again and found a correlation exists between the TyG index and insulin resistance, as insulin resistance weakens the body's response to insulin, impacting the liver's ability to process glucose and fat metabolism. In cases of insulin resistance, the liver may increase triglyceride production while decreasing glucose utilization, leading to elevated levels of triglycerides and blood glucose in the bloodstream. Oxidative stress triggered by high blood glucose levels can exacerbate the inflammatory response in acute pancreatitis (AP) and serve as a standalone prognostic indicator for AP. Additionally, elevated triglyceride levels can worsen pancreatic ischemia, hypoxia, microcirculation abnormalities, and systemic inflammatory response, all of which can complicate AP. Therefore, an elevated TyG index can serve as an indicator of insulin resistance. We have mentioned this in the third paragraph of Discussion marked in yellow and reference [31,32,33,34] has been added.

**Comment** **3:** *Lack of Continuous Monitoring**: Evaluating the TyG index at a single point in time may represent a more severe clinical situation for one patient compared to another. Therefore, I suggest that the analysis be dynamic to assess the real effect on the patient's metabolic state and its impact on mortality.

**Response:** Due to the limitations of the MIMIC-IV database itself, we were unable to obtain dynamically changing fasting blood glucose and triglyceride levels. We tried to collect these two indicators in other time periods, but the data loss exceeded 50%, making it impossible to conduct rigorous statistical analysis. We hope the reviewer understands these difficulties.We have mentioned this in the last paragraph of Discussion marked in yellow.

**Comment** **4:** **Generalization of Findings**: The findings may not be generalizable to all populations. The study sample is specific to critically ill patients with acute pancreatitis, and the results may differ in other contexts or populations. Additionally, the etiology of pancreatitis needs to be better established.

**Response:** It is really true as Reviewer pointed out that the findings may not be generalizable to all populations, We have explained this shortcoming in the Conclusion marked in yellow. The MIMIC-IV database only includes patients with biliary pancreatitis and alcoholic pancreatitis, not common hyperlipidemia pancreatitis. This limits the accuracy of etiological data collection. Making hasty generalizations could result in the loss of valuable clinical data and authenticity. Our center is currently conducting a prospective study to gather data on acute pancreatitis cases from various causes. We anticipate that latter data analysis will provide statistically significant results.

**Commen**t **5:** **Variability in Laboratory Measurements**: Variability in laboratory measurements, fasting times, whether the patient has a patent oral route, and other methods such as the timing of sample collection can affect glucose and triglyceride levels, impacting the accuracy of the TyG index. This needs to be better established.

**Response:** It is really true as Reviewer pointed out that variability in laboratory measurements, fasting times, the timing of sample collection can affect glucose and triglyceride levels, impacting the accuracy of the TyG index. We also noticed this flaw. Due to the limitations of the MIMIC-IV database,we were unable to obtain the specific times at which fasting blood glucose and triglycerides were measured.Blood glucose and triglyceride measurements were not standardized for each patient, different therapy and food intake may also affect the frequency of blood glucose and triglyceride measurements. We have mentioned this in the last paragraph of Discussion marked in yellow.

**Comment 6:** **Disease Severity Factors**: Factors such as disease severity, measured by the APACHE II score (Acute Physiology and Chronic Health Evaluation II), were not fully considered. These factors can significantly impact the study's findings, representing an important bias in this work.

**Response:** Some confounding factors, including metabolic syndrome parameters,Acute Physiology and Chronic Health Evaluation II (APACHE II), nutritional state parameters, and infammatory markers, were not thoroughly considered. It is a pity that there is no score for APACHE II recorded in the MIMIC-IV database.This may have an impact on the results. Our center is currently conducting a prospective study to gather data on score for APACHE II. We anticipate that latter data analysis will provide statistically significant results.

Dear Editor and reviewers,We hope that the revision is acceptable, and your favorable consideration of our manuscript is greatly appreciated. Best regards.

Jian Liao

2024-05-23

Discussion marked in red.e mentioned this in the last paragraph of Discussion marked in
